# Proposal for an initial screening method for identifying microplastics in marine sediments

**DOI:** 10.1038/s41598-021-00228-3

**Published:** 2021-10-19

**Authors:** Toshiro Hata, Ningjun Jiang

**Affiliations:** 1 Department of Civil and Environmental Engineering, Graduate School of Advanced Science and Engineering, Hiroshima University, 1-4-1 Kagamiyama, Higashi-hiroshima, Hiroshima, 739-8527 Japan; 2 Institute of Geotechnical Engineering, Southeast University, Nanjing, 211189 Jiangsu China

**Keywords:** Environmental economics, Environmental impact, Civil engineering

## Abstract

Marine debris, which is often called microplastics, is widespread in marine environments, particularly in sediments, and is recognized as an environmental hazard because it concentrates contaminants, forms biofilms, and sinks into marine sediments. In sediments, it may be ingested by benthos and have a negative impact on higher food chain levels. In this study, a new protocol was developed to identify microplastics in various sediment fractions. This protocol combined sieving and staining based on ordinal geotechnical/geological testing methods. The sieving process was derived from the conventional particle size distribution test, and nontoxic dyes were employed in the staining process. The protocol is safe and easy to perform as it merely involves the use of conventional geological/geotechnical testing equipment. The new protocol was successfully employed to stain and categorize different types and sizes of microplastic particles from contaminated sediments. This safe, easy-to-use, and efficient protocol can serve as the basis for a new alternative approach to study microplastics present in sediments, which can be performed using basic materials familiar to geotechnical/geological engineers.

## Introduction

The 17 Sustainable Development Goals (SDGs) listed in the 2030 Agenda for Sustainable Development by the United Nations has urged the scientific community to promote a better understanding of these topics. SDG #14 encompasses targets that consider the marine ecosystem, including marine debris. One type of marine debris, classified as microplastics, floats on the sea surface, is deposited in deep sea beds, or is stranded on the coastline, posing environmental risks to marine biota^1,2^. These microplastics can concentrate toxic chemicals such as organic compounds, persistent organic pollutants^3,4^, and trace elements^5,6^ and can further increase the ecotoxicological risks from sediments. Previous research has reported that microplastics are widely deposited in shallow-to-deep seabeds. Woodal et al. reported that deep seabed sediments include microplastics that are 2–3 mm in length and < 0.1 mm in diameter^7^. Alomar et al. reported that shallow sediments include microplastics that are 0.063 mm to > 2 mm in diameter^8^. Because of their small sizes, microplastics are ingested by zooplankton and are transferred to higher food chain levels, thereby becoming harmful to marine ecosystems^3,9^. SDG #14.2 focuses on achieving healthy and productive oceans, which includes evaluating the environmental impact caused by microplastic debris present in marine sediments.

Microplastics are classified into five categories based on their source: (1) direct manufacturing such as facial cleansers^10^, (2) subdivided or fragmented large plastic debris that has undergone degradation after exposure to the ocean environment^11^, (3) microfibers and textiles from garment laundry^12,13^, (4) synthetic rubber particles released from car tires^14^, and (5) disposable plastic products such as food containers and an increased production and usage of surgical face masks due to the COVID-19 pandemic^15^. Microplastics can contaminate sediments in coastal areas with high population densities^9^. Microplastics such as polyethylene (PE), polypropylene (PP), polystyrene (PS), and polyamide (PA) are commonly found in river sediments^16^, and PP, PE, and polyvinylchloride (PVC) are abundant in marine sediments^11^. PE, PP, and PS are industrial products that can spread across the sea surface easily because of their physical characteristics such as low density. Moreover, combined with natural particles such as clay, they can form biofilms. The accumulation of microorganisms on microplastics in biofilms may increase their density, accelerate their vertical transport, and cause them to sink into benthic sediments^17^.

To determine the environmental impacts of different types of microplastics in aquatic habitats, they should be isolated and identified. Microplastics or microfibers present in sediments are usually identified via specific gravity separations, followed by microscopic observations^18^. However, although isolation of microplastics via specific gravity separations can be applied to types of plastics over 1.20 g/cm^3^, the identification of small particles and fibers using microscopes can be challenging and inefficient^19,20^. Fourier-transform infrared (FTIR) or Raman spectroscopy combined with microscopy are often used to study the polymer types of microplastics or microfibers, and thermogravimetric/differential thermal analysis (TGA/DTA) is used to identify the types of plastics^21^. However, operating these systems requires a trained technical staff with expertise in chemical analysis. To address this issue, the use of lipophilic dyes that enable the visualization of microplastics or fibers using fluorescent microscopy are recommended^22,23^. The advantage of this approach is that it does not require expensive analytical instruments such as FTIR and Raman spectroscopes. However, a fluorescence microscope should be used to visualize microplastics, but it is only applicable to the study of floating microplastics and those mixed with organic materials. It has low efficiency for clay-coated or sediment-contaminated microplastics. However, some researchers have developed protocols for extracting microplastics from marine sediments^24–27^.

**Figure 1:**
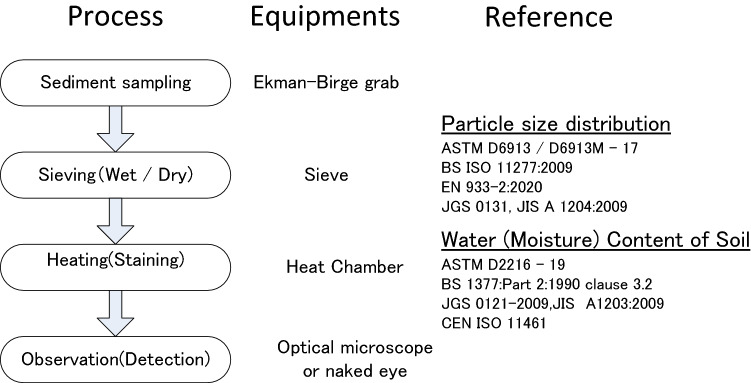
Schematic diagram of the proposed method.

Coppock et al., proposed a revised method for separating microplastics and other particles using density flotation, which can be used to recover high-density microplastics from sediments^28^. However, this system requires zinc chloride, which has been classified as a toxic chemical^29^. In the present study, we developed a safer and easier protocol for identifying sediment microplastics using conventional geological and geotechnical testing equipment with an optical microscope that is also in compliance with standardized sieving procedures from several industrial standards (Japanese Geotechnical Society [JGS]^30,31^, International Organization for Standardization [ISO]^32,33^, European Norm [EN]^34^, and the American Society for Testing and Materials [ASTM]^35,36^). The framework of this newly proposed method is shown in Fig. 1.

The proposed method uses basic equipment generally used for soil classification tests (particle distribution and moisture content tests) combined with a standard optical microscope used in geotechnical or geological fields. Moreover, this method does not require the use of toxic chemicals for staining the plastics and does not require extensive training. When combined with the sieving process, the microplastics in each sediment fraction could be separated and clearly identified.

The proposed method is highly efficient for detecting clay aggregate microplastic particles covered with biofilms and can detect the vertical distribution of microplastics from core samples and evaluate the relationship between particle size and adsorption or capture ability. Moreover, it can be performed using conventional (standard) laboratory instruments available at soil testing companies or educational institutions within 90 min. This method can also promote environmental risk control with soil science or engineering activities and encourage education for younger generations, facilitating the achievement of the SDG 14 goals. In this article, we elaborate on the proposed method and demonstrate the results of its application on actual marine sediments collected at Shin-minato port at Toyama, Japan, to identify common types of microplastics.

## Results

### Staining color and staining of different plastic types

**Figure 2:**
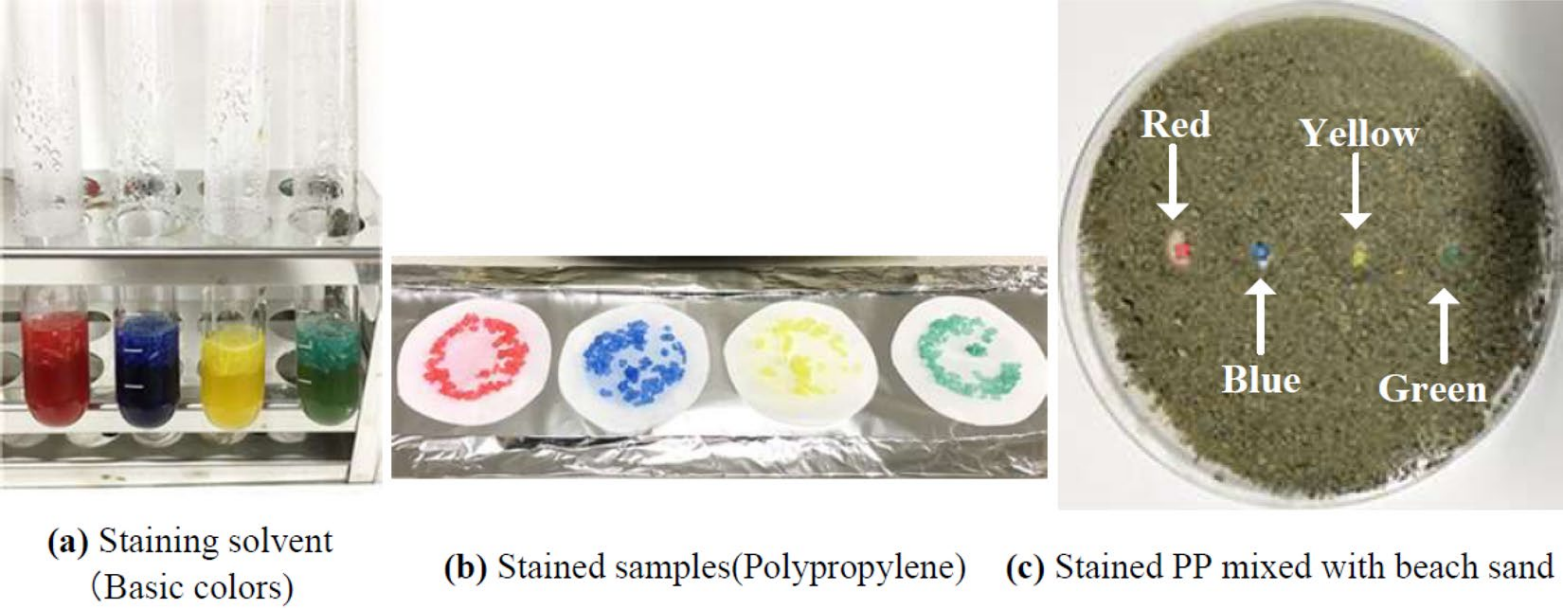
Results of staining with (**a**) undiluted staining solvent for polypropylene (PP), (**b**) 1:10 diluted staining solvent for polypropylene (PP), polyethylene (PE), and polystyrene (PS), (**c**) Stained PP mixed with beach sand.

PP particles were stained at 105 °C for 20 min using four base staining solvents (Fig. 2a). Undiluted staining solvent was used in the procedure. The four samples stained with different-color stains were collected on quantitative filter paper (retained particle size of 4 µm) using a suction filtration machine, and the staining effectiveness was evaluated via visual observation (Fig. 2b). All PP samples were stained with four non-diluted staining solvents of different colors and were easy to identify with the naked eye. However, when PP particles were mixed with sediments, most of the sediment particles stained dark, which can potentially hinder particle identification. The identification of stained microplastics mixed with natural sea sand is shown in Fig. 2c. The red-stained microplastics mixed with beach sand were easy to identify visually compared with yellow-, green-, and blue-stained microplastics.

**Figure 3:**
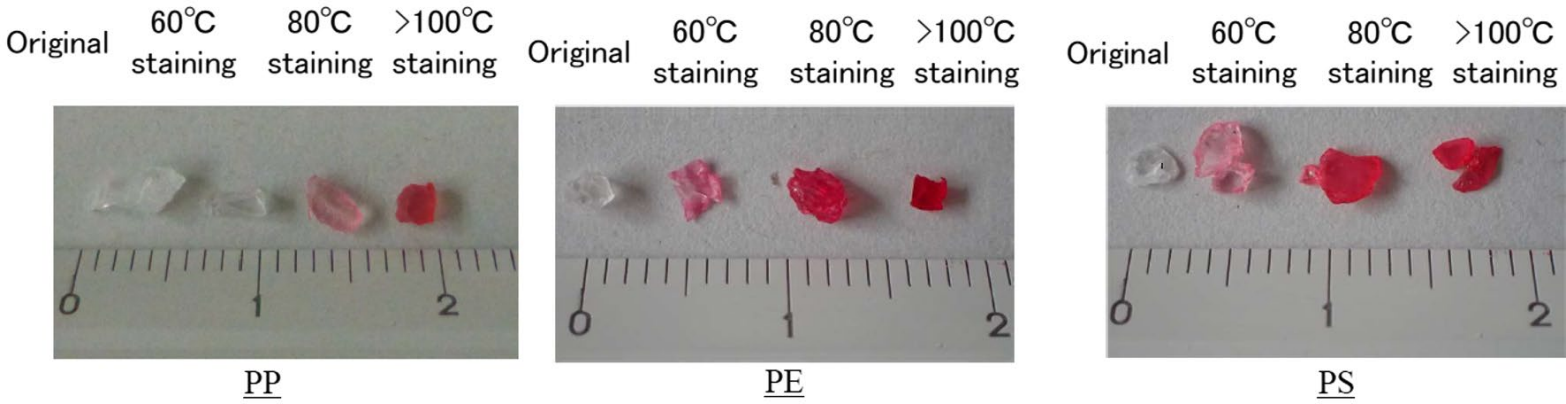
Results of the proposed method for staining at 60 °C, 80 °C, and 105℃ for 20 min.

In the second experiment, we evaluated the possibility of roughly classifying the plastic species according to the staining temperature. The relationship between the types of plastic and staining temperature under atmospheric pressure conditions is shown in Table 1. The results of the proposed method of staining the samples at temperatures of 60 °C, 80 °C, and 105 °C for 20 min are shown in Fig. 3. All types of plastics could be stained, and the increase in staining intensity depended on the staining temperature. PE and PS were almost completely stained at the 80 °C condition. However, PP was almost completely stained only at the > 100 °C condition. These results suggest that the staining temperature can affect the staining intensity and be used to roughly classify the plastic species. The possibility of roughly classifying plastic species only based on their staining temperature (60 °C, 80 °C, and > 100 °C) without a chemical analytical machine is one of the advantages of the proposed method.

**Table 1:** Relationship between the type of plastic and staining temperature under atmospheric pressure.

Staining temperature	Type of plastic	Comment
Under 60 °C	PVC	
60 °C–80 °C	PE, EVA, ABS, and PS	ABS: Less than 70 °C
80 °C to boiling temperature	PA, PEs, POM, PP, and PC	

PVC: polyvinyl chloride, EVA: Ethylene–vinyl acetate, ABS: Acrylonitrile butadiene styrene, PEs: polyester, POM: polyacetal, PC: polycarbonate.

**Figure 4:**
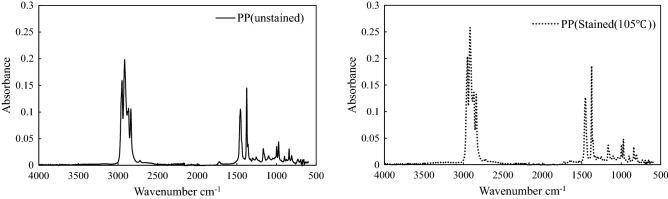
ATR spectra of unstained and stained PP.

In the third experiment, the influence of the staining process (chemical or thermal effect) for typical microplastics (PP) was investigated using attenuated total reflection–Fourier-transform infrared spectroscopy (ATR-FTIR) (FT/IR-6600, Jasco, Japan). The ATR-FTIR results are shown in Fig. 4. The ATR spectra of each sample were quite similar, and the proposed staining method was not affected by the ATR-FTIR analysis. The results show that the proposed staining method can retain the original state of the plastic without affecting its chemical properties and does not affect the results of additional analyses such as ATR-FTIR.

**Figure 5:**
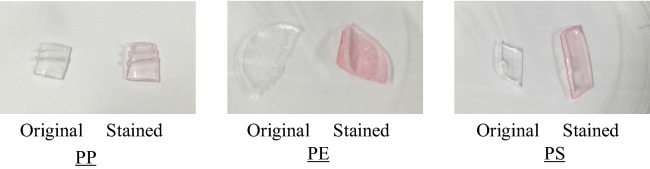
Results of staining with 1:10 diluted staining solvent for polypropylene (PP), polyethylene (PE), and polystyrene (PS).

In the final experiment, the staining results were found to be consistent across all three replicates for all fractions, where PP, PE, and PS were stained with 1:10 and 1:20 diluted staining solvents. The results of staining with the 1:10 diluted staining solvent is shown in Fig. 5. In the final experiment, the 1:20 diluted staining solvent sample was not bright, and it was difficult to identify the sand particle-contaminated sample. Moreover, the resulting colors were light compared with those of the first experiment (Fig. 2a). However, red color can be used to identify microplastics contaminated with natural beach sand using the naked eye and microscopic observations. However, other polymers such as polytetrafluoroethylene (PTFE) were not stained with the diluted staining solvents at the same conditions. PTFE requires high temperatures and high pressures for staining.

### Staining marine sediment samples using the sieving process

**Figure 6:**
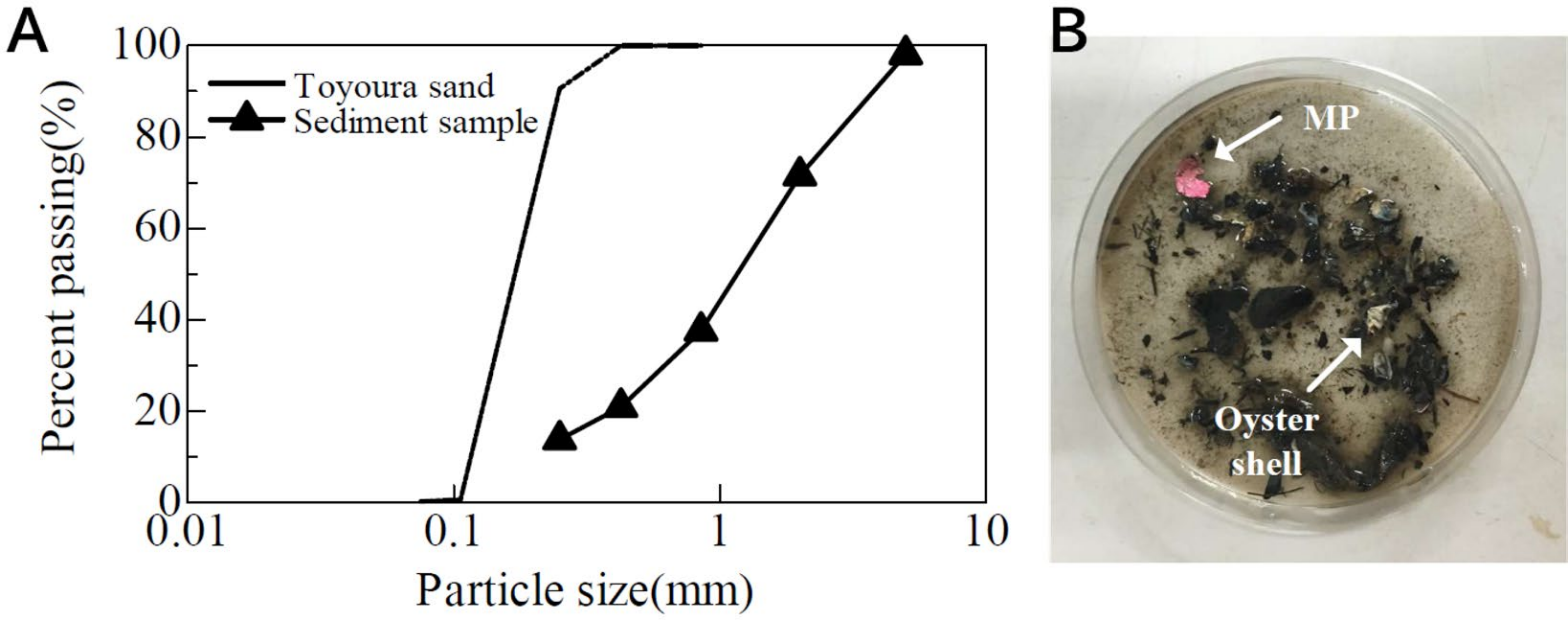
Results of staining with (**A**) undiluted staining solvent for polypropylene (PP), (**B**) 1:10 diluted staining solvent for polypropylene (PP), polyethylene (PE), and polystyrene (PS).

**Figure 7:**
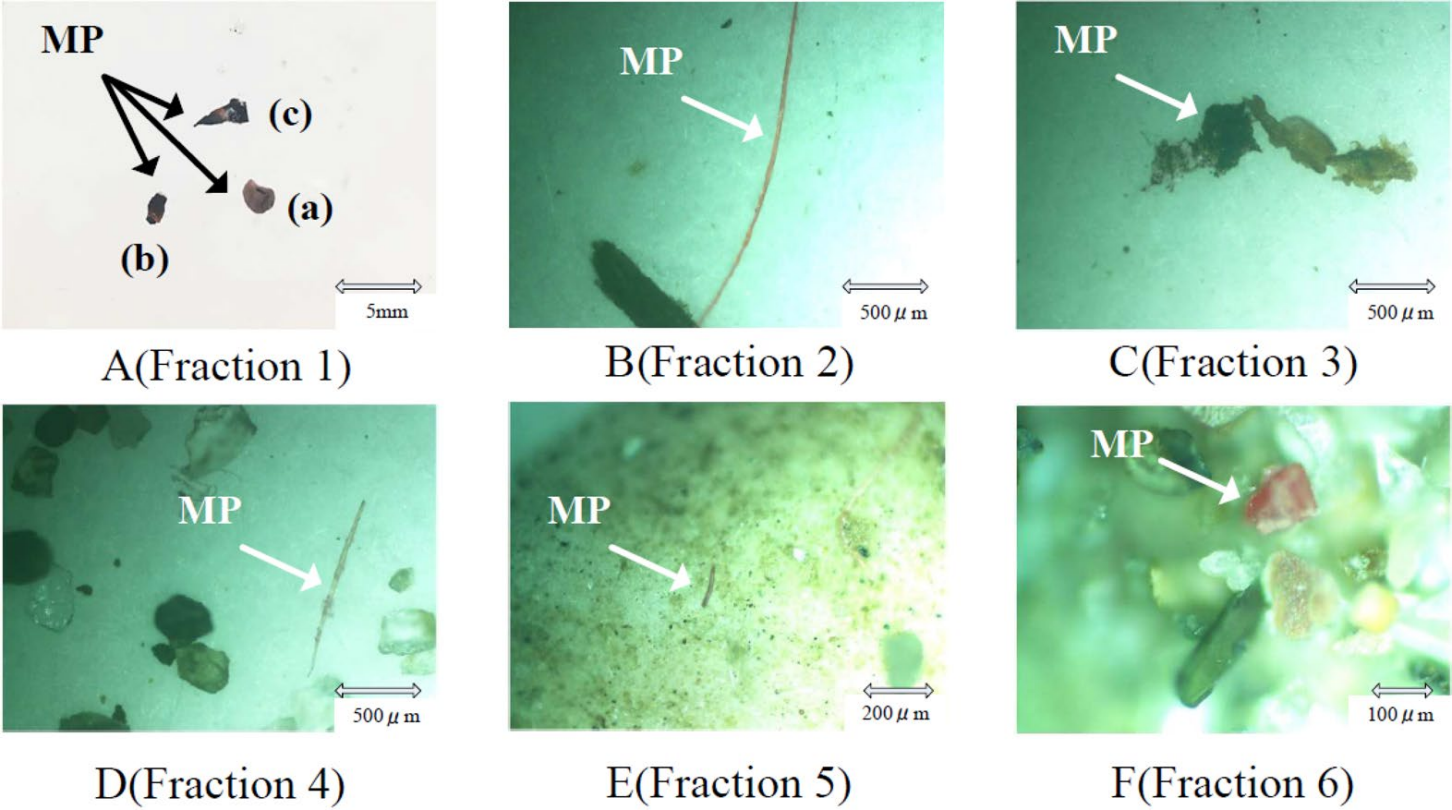
Microplastics (MP) from the sediment samples were stained and visually observed (fractions 1–6) unaided and using a microscope (fractions 2–6). (**A**) fraction 1, particles > 2 mm, photographed using an anα5100 Sony camera, (**B**) fraction 2, particle size 0.85–2 mm, (**C**) fraction 3, particle size 0.42–0.85 mm, (**D**) fraction 4, particle size 0.25–0.42 mm, (**E**) fraction 5, particle size 0.106–0.25 mm, and (**F**) fraction 6, particle size < 0.106 mm.

The particle size of dredged sediments in our study ranged from 0.106 mm to > 2 mm (mean [D50] = 1.16 mm). Figure 6A shows the particle distribution curve from dredged sediments and Toyoura sand (Japanese standard sand). The particles in the dredged sediments are larger than those in Toyoura sand (ranging from 0.1 to < 1 mm) possibly because the sediments at Shin-Minato port originated from oyster shells and other human-related wastes and are likely to contain a wider range of microplastics/fibers. The red color staining solvent stained the large microplastic particles (> 5.0 mm) in the sieved sediment sample (Fig. 6B). Many large particles are mixed with crushed shells coated with small substances such as clay or biofilms. The results of the staining of air-dried sediments (fractions 1–6) using our proposed method are shown in Fig. 7. The staining process generated small particles from substances coating the sample surface, which generally made the samples turbid and identification of the microplastics difficult. However, despite this turbidity, the proposed method can be used to easily identify plastics sized > 5 mm that are mixed with shiny materials such as shells (Fig. 6B).

The stained and unstained samples were analyzed using ATR-FTIR to classify the microplastics. The results of ATR-FTIR analyses indicated that the red-stained samples were PP. The white-stained samples could not be identified. These results indicate that the proposed method can be used to easily distinguish between plastic debris and inorganic substances.

The results of the microplastic classification using our proposed method are shown in Fig. 7A. The particles of fraction 1 (> 2.0 mm) could be visually identified and were photographed using a normal camera (α5100 Sony, Japan). Microplastics in this fraction originated from fragmented debris of daily necessities, such as medical press through pack sheets and styrene foams. To observe the particles of fractions 2–6 (Fig. 7B–F), a microscope (BHM series, Olympus Japan) with a charge-coupled device camera (EL310, Wraymer) was used. These fractions mostly contained small-sized particles such as fiber dust and crushed materials from daily waste and were detectable after staining (Fig. 7B–E). The particles of fraction 6 (< 0.106) were cube-shaped and stained red (Fig. 7F). The source of these small particles was identified as contamination from self-precipitated crystals from the staining solvent solution and was separate from the original microplastics or microfibers from the sediment.

**Figure 8:**
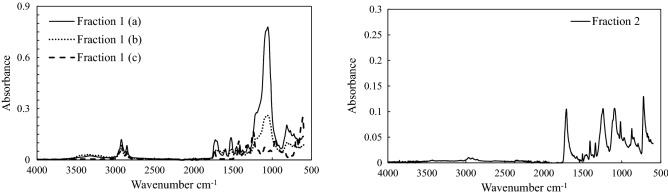
ATR spectra of fractions 1 and 2.

To evaluate the accuracy of the proposed method, ATR-FTIR analysis was performed to identify the plastic species stained in fractions 1 and 2. The results of the ATR-FTIR analysis are shown in Fig. 8.

The fraction 1(a) and (b) samples are classified as slow-release fertilizers. The slow-release fertilizer material is based on the composite of polyurethane and inorganic substances such as talc. The fraction 1(c) sample was classified as similar to PVC and included materials used in our daily lives. The fraction 2 sample, which included plastic fiber, was classified as PET, which is used in clothing fabrics.

All the samples stained using the proposed method were classified as a certain type of plastic, confirming the effectiveness of the proposed method.

The number of microplastics detected from the dredged sediment with sieving for each fraction is shown in Table 2. Almost the same numbers of plastics were detected irrespective of the fraction. This result indicates that plastics with a wide range of particle sizes are homogeneously deposited in shallow marine sediments.

**Table 2:** Number of microplastics detected from the dredged sediment samples.

Fraction number	Particle size (µm)	Number of detections
1	~ 2000	2
2	2000–850	2
3	850–420	3
4	420–250	3
5	250–106	2
6	106–75	5

## Discussion

Visualizing microplastics obtained from dredged sediments is vital for studying their impact on the marine ecosystem. Currently, low-cost and easy detection methods to study the accumulation of microplastics in the environment are available^37^. A tracing method that can be used to visualize fluorescent microplastics using a general industrial ultraviolet flashlight has been reported^38^. However, it can only detect a limited number of microplastic types and cannot be used to identify nonfluorescent microplastics in the environment. Moreover, conventional methods based on specific gravity separations use saline water, which hinders the separation of biofilm coatings or clay aggregate microplastics from actual dredged marine sediments.

Therefore, it is necessary to develop separation and identification methods that are nontoxic, easy to perform, and can efficiently detect many types of microplastics under natural conditions, which can be applied to contaminated microplastics without the use of any specific equipment.

In this study, we developed a technique to isolate, stain, and detect (and roughly classify) microplastics from sediment samples that are derived from the environment. This combined method that involved staining and sieving processes can be used to separate microplastic particles based on their size and type and is in compliance with geotechnical industrial methods and standards (JGS^30,31^, ISO^32,33^, EN^34^, and ASTM^35,36^).

The target of the proposed method is microplastic contaminated sediment which contains daily and agricultural use plastics such as chemical fertilizer, laundry drainage mixed with waste textiles, etc. This proposed method has several advantages. First, it employs basic geotechnical or geological equipment with nontoxic reagents, which are available in most geological engineering fields. It can also be combined with microscopy, enabling the detection of small-sized microplastics. Second, it can be applied to ground and soil contamination and other construction and environmental industries during implementation in industrial projects. Third, the proposed method can be completed in approximately 1 h.

### Staining solvent and staining of different types of plastics

It is important to select a suitable staining color solvent from commercially based anthraquinone and azo dyes for staining sediment and particle mixes. Azo dyes can stain both natural and synthetic textile products, and their staining procedure is simple^39^. In this study, we investigated the efficiency of the staining color solvent under different dilutions and showed that diluting the solvent leads to a weaker staining result. Moreover, red-stained microplastics mixed with sediments were more visible than microplastics stained with other colors; therefore, we used the red stain in our experiments. The solubility of the dye is an important parameter in staining^40^, and our findings show that it is essential to adjust the concentration of the staining solvent for various plastics types before the experiments and field application.

The testing procedure of the proposed method is as follows: (1) wet sieving processes are applied to dredged sediments using standard methods (JGS, ISO, ASTM, EN, etc.), (2) red-colored staining solvent is diluted tenfold with distilled water and filtered through a 0.25-mm membrane, (3) the sieved sediments are mixed with diluted staining solvents and heated to 105 °C for 20 min, and (4) after cooling, the sample is visually observed unaided and also using an optical microscope.

The azo dye-based staining solvent can be used to roughly classify different types of plastics according to the staining temperature. The relationship between the type of plastic and staining temperature under atmospheric pressure conditions is shown in Table 1.

The possibility of roughly classifying plastic species based on only their staining temperature (60 °C, 80 °C, and > 100 °C) without a chemical analytical machine is one of the advantages of our proposed method.

However, our method failed to stain fluoride polymer microplastics, indicating that our protocol may not be successful in detecting all types of microplastics under atmospheric pressure. We could stain PTFE under high pressures and temperatures that are similar to autoclave sterilization conditions (121 °C and 0.07 MPa), but the result was not reliable due to the irregular staining pattern.

### Staining field sediment samples using the sieving process

Identifying small particles is important in the study of marine ecosystems because those are available to benthic microorganisms and can be transferred to higher food chain levels through ingestion^3^. The proposed method can be used to detect microplastics such as those with biofilm coatings or aggregates with other substances from dredged sediments more easily than other available techniques. ATR-FTIR analysis could identify the red-stained microplastics (PP) but failed to detect white-stained particles. This positive result implies that the proposed method can be used to visually classify the types of microplastics, contrary to the other available methods of microplastic observation^41^. Moreover, the smaller particles in fractions 2–5 were stained successfully and more efficiently than that using other tracing methods such as Nile red and fluorescence staining^37,42^.

The proposed method combined with sieving and staining can be used to selectively identify different types of plastics from seabed sediments using a few simple operations.

## Conclusions

In this study, microplastic-contaminated sediments close to the mouth of rivers or estuaries were examined using a combination of a new staining procedure and conventional geotechnical equipment. This proposed method uses a nontoxic azo dye that is safe and easy to apply and can be used by geotechnical/environmental engineering firms. It can also be taught to students in primary elementary and junior high schools. Laboratory tests using artificial plastics mixed with Toyama sand indicate that the best staining solvent color is red. When actual dredged sediments were tested, the proposed method allowed unaided visual detection of large-sized nano/microplastics and the detection of smaller particles with the use of an optical microscope. In summary, wet sieving combined with staining can easily distinguish microplastics in sediments. Moreover, particles larger than 2.0 mm can be observed with the naked eye, even when the sample is turbid, and particles ranging from 0.1 to < 2.0 mm can be observed using a microscope. The method can also distinguish particle types. This study is significant because a method that is affordable, easy, and can efficiently analyze microplastics in sediments was developed. However, additional experiments under various temperatures and with various heating durations are required to evaluate this method to determine optimal staining conditions. In addition, we will use sediments collected from a variety of geographic locations and from different depths to further test the efficiency of our method. Finally, we will combine artificial intelligence screening methods with the proposed method of staining photos to identify the microplastics based on shape and color information. The final goal of this research is to establish easy screening methods without the use of specific instruments such as TGA-DTA and ATR-FTIR and promote primary or secondary grade environmental education.

## Materials and methods

### Staining color and staining of different plastic types

A staining solvent using nontoxic anthraquinone and azo-based disperse dyes was used in the experiment (Murakami Corp, Kyoto Japan). This solvent included the four colors (yellow, red, blue and green, Fig. 2a). First, the major microplastic source, PE, was stained with the basic four colors, which were checked with staining intensity without soils. The four colored PP samples were mixed with natural beach sand from Toyama Bay, Japan, and their visibility was evaluated. In the second experiment, three types of plastics were included in the staining process with undiluted red staining solvent at three temperatures, and the effect of the staining temperature was assessed, which can be used to roughly classify plastic species. The optimal staining temperature of the three types of plastics is shown in Table 1. The classification of microplastic species with the staining process depends on the staining temperature: 60 °C to 80 °C for PE and 80 °C to > 100 °C for PP and PS. In the second experiment, a heat block (dry bath) machine (HDB-2N, As one, Japan) set to three temperature conditions was used to heat the samples for 20 min.

The effect of the proposed staining method was assessed using ATR-FTIR, a conventional microplastic detection method. Two samples, i.e., the original sample and 105 °C-stained PP sample, were evaluated using ATR-FTIR (FT/IR-6600, Jasco, Japan).

Finally, the selected staining solvent color is red that applied the recommended dilutions, 1:10 and 1:20 for this experiments, respectively, to stain PP (specific gravity = 0.91), PE (specific gravity = 0.92) and PS (specific gravity = 1.05) pellets (Sanplatec Corp., Japan). The selected staining solvent can selectively stain various microplastics, including PP, PE, and PS, under multiple staining temperatures. Three types of microplastics were selected to determine the efficacy of the staining process. In the final experiment, a heat block (dry bath) machine (HDB-2 N, As one, Japan) set to a maximum temperature of 105 °C was used to heat the samples for 20 min, and the staining efficiency was determined using the naked eye, which will define the reasonable dilution rate of the proposed method such as non-dilution, 1:10 or 1:20.

### Staining field sediment samples using the sieving process

A sediment sample was collected using an Eckman barge bottom sampler (Rigo, Japan) from Shin-Minato port yard at Toyama, Japan (GPS coordinates: N36°46′15.8″, E137°05′46.8″; depth from sea surface: 2.8 m).

The sediment sample was sieved into six fractions: (1) > 2.0 mm, (2) 0.85–2.0 mm, (3) 0.42–0.85 mm, (4) 0.25–0.42 mm, (5) 0.106–0.25 mm, and (6) < 0.106 mm. Three replicates were analyzed from each fraction. Fraction 1 (> 2.0 mm) was rinsed with tap water and placed in a 50-mL glass beaker. The beaker was filled with staining solvent to cover the top of the sample and was kept at 105 °C for 20 min. The applicability of the proposed method was validated by analyzing stained samples from fractions 1 and 2 using ATR-FTIR analysis (Nicolet Summit, ThermoFisher), and the plastic species were confirmed. In ATR-FTIR, an infrared spectra database is used to accurately determine the type of plastic^43^.

The remaining fractions were used in the following steps. We placed 0.2 g of each fraction sample in individual 15-mL glass test tubes. Then, 10 mL of tenfold diluted red staining solvent was added to each test tube and mixed with the sample using a vortex mixer (Kenis, Japan). After mixing, the test tube was covered with an aluminum cap to prevent contamination with microfibers from the room air. Staining was conducted at 105 °C for 20 min. Immediately after the heating process, the samples were rinsed with tap water three times. The samples were rinsed gently and carefully to avoid losing the floating types of microplastics. At the end of this process, the dye remaining on the microplastic surface was used for additional analyses. Rinsed microplastic mixed sediments were filtered using glass fiber filters with a vacuum filtration machine. After the vacuum filtration, the glass fiber filters were observed under an optical microscope (BHM series, Olympus Japan) to detect the microplastics or fibers.

## Data Availability

All data generated or analyzed during this study are included in this published article.
